# Supplementary material for: Cervical lesion proportion measure using a digital gridded imaging technique to assess cervical pathology in women with genital schistosomiasis
Source: PLoS Negl Trop Dis. 2022 Jul 5;16(7):e0009995. doi: 10.1371/journal.pntd.0009995 (PMC9286223; doi:10.1371/journal.pntd.0009995)
Supplement: Attachment — Submitted filename: Responses and description of changes.docx [file pntd.0009995.s001.docx]

*Reviewer's Responses to Questions*

*Key Review Criteria Required for Acceptance? As you describe the new analyses required for acceptance, please consider the following:*

*Methods*

*Are the objectives of the study clearly articulated with a clear testable hypothesis stated? Is the study design appropriate to address the stated objectives? Is the population clearly described and appropriate for the hypothesis being tested? Is the sample size sufficient to ensure adequate power to address the hypothesis being tested? Were correct statistical analysis used to support conclusions? Are there concerns about ethical or regulatory requirements being met?*

Reviewer #1: Major Comments:

1)	Validation: I believe a validation study would compare a new technology against a “gold standard” to confirm that the new technology accurately measures what it claims to measure. Admittedly, there is not a consensus reference standard for FGS. However, was there a comparison made with another accepted diagnostic method for FGS (biopsy, PCR, etc)? If there was no comparison with PCR or biopsy (or Holmen’s colorimetric image analysis), I’m not sure it is correct to call this a validation study. Please rephrase the title and line 301. Please also acknowledge this in the limitations.

We agree with the reviewer. A procedure of validation as such has not been performed and by which our new technique/method has been compared with another existing reference. In recognition of this situation, we have decided to leave “validation” out of the amended title. Hence, the title has been changed from “Validation of c*ervical lesion proportion measure using a gridded imaging technique to assess cervical pathology in women with genital schistosomiasis”* to “*Cervical lesion proportion measure using a gridded imaging technique to assess cervical pathology in women with genital schistosomiasis”*. Moreover, the term “validation” has also been removed from the manuscript text. Finally, this element of testing inadequacy has been added to limitations Line 457-463.

**The lines marked in red are referring to the document: “FGS Manuscript – with changes 5.3.2022”**

2)	What proportion of women in the study had STI? Please see PMID: 15772328. In Kjetland 2005, FGS-associated lesions (the homogeneous yellow sandy patch) were associated with STI (please see Table 4 in Kjetland 2005). There was strong evidence that chlamydia, HSV, and high risk HPV were associated with the homogeneous yellow sandy patches. Thus, to ensure that the lesions described were not associated with STI, please describe what STI testing was performed and what proportion of the women with sandy patches also had STI.

All participants in the RCT, including the female participants from which the digital images have been randomly selected for the purpose of the present study, have tested for STIs. Because of the covid19 pandemic the study team has encountered significant delays regarding flight transport of study samples from Madagascar to Denmark, and further to Germany, Holland, and Japan. Moreover, administration of the GDPR protocol has also added additional delay in the overall process in obtaining approval of exchange of data and biological samples between our research center in the North Denmark Region to the collaborating laboratory at Statens Serum Institute in Copenhagen in another region of Denmark due to different perceptions of the regulations, and due to lack of available staff in the legal departments working on other non-covid19 related tasks, which have been down-prioritized. However, it is our hope that we will have the results of STI, Schistosoma DNA PCR and other analyses available by 2023, and by which the results of gridded imaging technique will be presented in the overall context of complete FGS and STI diagnoses in conjunction with the RCT publication. So far, the aim of this study is to describe the development and application of the new gridded imaging technique.

3)	Please could the authors describe any assessment for dysplasia or malignancy? Neovascularization (see Kjetland 2005 Table 5) has been strongly associated with CIN I-III). Thus, in lines 114 & 116 these sentences could say “in women with FGS-associated lesions” or some other language to inform the reader that we do not know with certainty that these lesions are due to FGS, rather only that they look like lesions that are associated with FGS.

The results of PAP smears are also pending and will be reported later.

We agree with the reviewer regarding the uncertainty of lesions and origin/etiology. See next item 4)

4)	Given that FGS lesions lack specificity, I think it is challenging to call these “pathognomonic” lesions. Please rephrase throughout. Additionally, without an evaluation for STI + malignancy + other diagnostic methods for FGS, it seems appropriate to use less definitive language around causality of these lesions by FGS (see lines 36 (fgs-evoked), line 42-43 “women with FGS”, line 116 “women with FGS”, line 130). However, in some locations the authors are examples of more measured language, suggesting association rather than causality. For example, at line 35 the authors say that “Female genital schistosomiasis (FGS) is characterized by a pattern of lesions”. I think this type of language throughout would be more appropriate.

We have rephrased “ FGS pathognomonic lesions” to “FGS associated lesions” throughout the manuscript. However, we believe that rubbery papules should be considered a pathognomonic lesion.

5)	Abstract: (Line 38) You say that “no tools are able to quantify clinical pathology at the cervix in a standardized and reproducible manner”. However, in the discussion you reference Holmen’s colorimetric image analysis. Is that tool not standardized and reproducible? Also Holmen’s method can also be applied to digital images.

It is correct that colorimetric image analysis (CIA) can be applied to digital images. However, in the paper by Holmen et al (Medical Engineering and Physics 37 (2015) CIA was claimed to be a diagnostic tool in FGS, whereas it was not stated as being applicable for quantification of cervical pathology.

6)	Methods:

a.	Line 159 – 164. It seems to me from these lines that the computer algorithm automatically counted and marked the FGS associated lesions (so the computer algorithm calculates the CLP)? If this is true, what was the role of the human reviewers? In line 214 it appears the reviewers also calculate a CLP – how is this done? Without a clear understanding of the human component, it is challenging to understand how meaningful the inter-rater reliability is.

We apologize if the text appears unclear. Basically, the observers (=human reviewer) review/evaluate the digital images systematically, square by square, in search of FGS associated lesions. If a square of the 424 equally sized squares in the grid contains at least one type of a lesion, then the observer will mark this square by use an marking arrow navigated from the computer keyboard as he or she will mark all the squares containing lesions among the 424 squares. The total number marked squares will then successively be counted in simple manner by the FGS Qubifier software program and the final proportion will then be presented on the computer screen (marked with a red arrow in Figure 1).

We have now amended the text aiming to make the content clear. First of all the text has been amended in the Methods (*Gridded imaging technique and cervical lesion proportion*). Line 209-228. In other parts of the text, the essential role of the observer in assessing the lesions has been added, hence making it more clear that identify the cervical lesions positioned in the squares in the grid. Then the final number of marked squares are added up by the program, which also translate the total number of marked squares into a final CLP value (in percentage)

b.	Lines 165-167 – to clarify, if there were 25 lesions/squares, this number 25/424 = 6% coverage of the cervical surface? These sentences might need to be rephrased slightly - this information was challenging to access.

We agree. The sentences have been rephrased. Moreover, we have also added an example on how the CLP is estimated by the FGS Qubifier software program based on the number of squares being identified by the observer as containing FGS associated lesions (25/424 x 100 = 5.8% (6.0%). Line 228-230.

c.	Line 188 - What are the two components of the experience profile (1+1) – is one number for gynecology and one for FGS? This should be clarified, and how does one ‘achieve’ minor/moderate/major experience? This seems slightly arbitrary and should be further explained and disclosed as such (as you did very nicely with the grading of lesions into mild/mod/severe).

We agree. It may seem a little confusing. We have therefore changed the description of the experience levels (gynecology in general and FGS, respectively) for three observers. We hope that will work better. Line 252-600

Minor Comments:

1) Lines 178 - Types of lesions. I think there is some debate regarding whether neovascularization should be included as an FGS associated lesion (in PMID: 25412334 the authors note that only homogeneous and grainy sandy patches, along with rubbery papules meet the criteria for FGS based the result of an expert meeting in 2010). I acknowledge that all 4 FGS-associated lesion the authors describe are displayed in the pocket atlas, thus making all 4 reasonable to include. However, it might be nice to have a sentence in the discussion outlining the controversy.

We agree. We have therefore changed to first sentence in this paragraph to: The following four types of lesions are occuring in FGS^14,20^ and displayed in the WHO FGS pocket atlas.^13^. Hence, deleted “pathognomonic lesions” Line 237-238. Moreover, we have also added text in the Discussion addressing this issue. Line 384-390.

2)	Referencing

a.	Line 82 – epidemiology (Africa and the middle east) – please reference

Reference has been added. Line 119.

b.	Line 83 – how does *S. haematobium* affect the genital organs? Please expand and reference.

This information has been added with a reference stated. Line 119-124.

c.	Line 64 – please reference these complaints. There is only one study I am aware of in which dyspareunia is associated with FGS (PMID: 21572820)

In accordance to WHO dyspareunia is listed as one of different symptoms associated with FGS (<https://www.who.int/activities/increasing-awareness-on-genital-manifestations-of-schistosomiasis> ). However, dyspareunia has been left out, and genital itch plus pelvis discomfort added. Line 66 + 93-94.

d.	Line 66 (Author Summary). I think it is an overstatement to say that “FGS is considered as a major risk factor for transmission of HIV” – please rephrase

We agree. The sentence has been rephrased (major has been left out). Line 68.

e.	Line 89. I think it is an overstatement to say “FGS associated with increased risk of HIV acquisition”. There has only been one study suggesting an association with HIV acquisition and this was a retrospective study in women with Shaematobium antibodies (they did not have FGS evaluations) (PMID 30543654). Please rephrase.

We agree. The sentence has been rephrased (HPV left out). Reference 10 (Feldmeier et al, 1996) raises the hypothesis of a linkage between HPV and FGS. Line 96-99.

f.	Line 90 – While it is a compelling hypothesis, I am unsure that we have evidence to claim that FGS increases the risk of HPV infection. The references you have cited do not support this statement (#9-11). Please amend this statement or provide revised references.

Same as comment to e.

g.	Line 87-88. Please elaborate on which concrete symptoms or features of FGS (?dyspareunia, discharge, genital itching) might lead to a “poor quality of sexual life”.

Points well taken. The sentences have been amended. Line 92-94

3)	Other minor comments:

Line 38 (abstract): “A tool for quantification of the lesions is needed to improve FGS research and control programs” – would the tool also be useful for evaluating treatment?

Yes. We have added this perspective. Line 53-54. Moreover, the digital gridded imaging technique is applied in the RCT FGS study (evaluating efficacy of a high dosage PZQ treatment regimen) to which we have referred in Methods.

Line 70-72 – How does your tool allow for treatment at the community level? Would your tool also allow for monitoring of lesion resolution (as well as lesion development)?

Yes, we see that as a potential purpose of the digital gridded imaging technique. Sentence has been rephrased. Line 70-74.

Line 122 – should RCT stand for “randomized controlled trial”?

Yes, has been corrected. Line 135-136.

Line 127 – do you mean that the area highly endemic for *S. haematobium* (with a high prevalence of FGS?)?

Yes, has been corrected. Line 141.

Line 142 – what is the digital part of the study – do you mean the image review?

The term digital applies to 1. *digital* images and 2. *digital* counting of the squares in the grid containing FGS associated lesions, which have been marked by the observers manually (***non-digital***), 3. *digital* estimation of the cervical lesion proportion (CLP). We have now amended the text in the Methods aiming to explain and describe the principles in digital gridded imaging technique in hopefully a more clarifying way. Line 176-199.

Line 346 – would there be a role for a mobile phone colposcope here as well?

Yes, absolutely. Most likely for a diagnosis of FGS in a dichotomous manner (yes/no). For quantification of lesions it most likely requires use of a larger computer/portable screen, in particular if the lesions need to be categorized in the four subgroups (homogeneous and grainy sandy patches, rubbery papules in addition to neovascularization), as additional information to the CLP. See Line 410-412.

Reviewer #2: The objectives are clear: This study aimed to develop and validate a cervical lesion proportion (CLP) measure for quantification of cervical pathology in FGS.

The RCT study design has been clearly explained.

The sample selected from the population needs further explanation. There were 116 women enrolled in the RCT between 15-35y with FGS pathognomonic lesions. How were 70 of the 412 images selected? For the comparison between the observers, why were 40 images selected from weeks 5 or 10 and two images from each of 10 women at both visits? Which women were selected, and why?

We agree. The description on how and why the specific images were chosen is poor.

Images from week 5 and 10 were chosen since two of the reviewers, before initiation of the present study, already had scored images from week 0 and 15. Therefor blinding could not be fully secured. The 70 + 10 images were randomly selected and anonymized by the a computer program (RStudio). The images were chosen so that the number of images from both visits were equal and during the random selection of images 40 images came from different women whereas 20 images came from 10 women but at two different timepoints. Line 166-174

*Results*

*Does the analysis presented match the analysis plan? Are the results clearly and completely presented? Are the figures (Tables, Images) of sufficient quality for clarity?*

Reviewer #1:

a. What proportion of each of the FGS-associated lesions did the authors find? This is important given that neovascularization is not uniformly considered to be FGS-associated.

An absolute relevant question. We agree that neovascularization is not uniformly considered to be FGS-associated. The distribution of the four different lesions will be presented in a following manuscript with focus on the outcomes of the RCT study in which efficacy of a high-dosage PQZ regimen is compared with standard regimen (40mg/kg). Moreover, data on STIs are also available for more detailed analysis of the findings, incl. *N.* gonorrhoeae, C. trachomatis, T. vaginalis, M.genitalium, HPV and HSV, will also be presented.

b. Please review for the reader again here which steps were 2a and 2b.

The steps have been explained. Line 232-238

c. It might be helpful to define inter-observer and intra-observer reliability for those readers who do not have a background with these methods

Intra- and inter-rater reliability has been further described in the method section. Line 262-263

Reviewer #2: The results are accompanied by figures showing the comparisons of the findings by the three different observers.

Please can the authors further elucidate the proportion of homogeneous sandy patches and grainy sandy patches found.

See reply above a) to reviewer 1

Since getting photographs of the cervix may be difficult it would be on interest to know if when only 20% pf the cervical surface was only partially depicted, when the similar results were found between the more and less experienced observers. Can the authors please comment?

We are not quite sure what is asked for here. We believe that the reviewer are asking whether the observers agreed more when the entire surface of the cervix was visualized. In 27 of the images less than 100% but more than 80% of the surface of the cervix was visible. We found no difference in agreement between the observers whether the entire surface was visible or not. When comparing images from the same woman for example in the research set-up of evaluating medical treatment it is crucial that the area represented in the image is the same before and after the treatment.

***Conclusions***

*Are the conclusions supported by the data presented? Are the limitations of analysis clearly described? Do the authors discuss how these data can be helpful to advance our understanding of the topic under study? Is public health relevance addressed?*

Reviewer #1: -I'm afraid from the way the methods are written that I don't fully understand the role of the human reviewers if the computer algorithm is calculating the CLP (please see above).

-Please see above for additional items for the limitations

We understand the reason for the uncertainty by the reviewer, probably because we haven’t explained the different steps in the digital gridded imaging technique adequately in the Methods. That we have done now.

Reviewer #2: The conclusions are supported by the data and they describe the limitations, but since a colposcope is the recommended tool, the authors need to consider a way forward.

The authors do not suggest a comparison study between use of the the colposcope (a heavy and expensive piece of equipment) and the digital image documentation approach which overcomes the shortage of colposcopic equipment. We would need information comparing the accuracy of the colposcope and the camera, to determine the accuracy/effectiveness of this new approach. Finding an effective solution to the diagnosis of FGS urgently needs such a study .

We agree. The digital gridded imaging technique provides an opportunity to obtain a GLP measure, which is not the case by use the colposcope procedure itself, because assessment of the cervical lesions as a proportion of the total cervical portio surface in a structured manner requires a grid with squares placed on the digital image using a high-resolution screen. However, the colposcope can still be if use for the purpose of the digital gridded imaging technique if a camera is connected to colposcope and digital images are kept for review after the pelvic examination has been performed. In that way, the colposcope (or a mobile phone with a camera and an app device) can be used to identify FGS associated lesions as a diagnostic supportive means. And if GLP measure is needed/requested then the digital gridded imaging technique can be used as an add-on tool.

Editorial and Data Presentation Modifications? Use this section for editorial suggestions as well as relatively minor modifications of existing data that would enhance clarity. If the only modifications needed are minor and/or editorial, you may wish to recommend “Minor Revision” or “Accept”.

Reviewer #1: 1) Line 190-199 – The authors have explained that the 70 images were chosen randomly – was this from a total of 412 total images from the study? Overall, I find it the numbers (70 from 212 or 412) and timepoints (the majority from week 5 but some from week 10 but from 50 women) hard to follow. Taking one randomly selected image per woman makes sense (line 198) but why then 2 from 10 women?

We agree that the description of the selection of images is hard to follow. Changes have been made. Line 166-174

This needs clearer explanation and perhaps Figure 2 needs a flow diagram to show the total number of participants, the time points, and images. This would allow further understanding of the risk of bias. The explanation given from lines 237-239 is easier to follow than the explanation used in the methods.

A new version of Figure 2 has been edited

Reducing stigma – I think we as scientists can do our part to reduce stigma through the language we choose in our manuscripts. Please consider rephrasing:

Yes, we agree. The expressions have been rephrased/deleted

a.	Line 93 “resource poor” Line 103-104

b.	Line 102 “in rural Africa” Line 113

c.	Line 308 “contaminated” water Line 362

d.	Line 346 “in an African context” Line 400

Reviewer #2: (No Response)
