# Supplementary material for: Cervical lesion proportion measure using a digital gridded imaging technique to assess cervical pathology in women with genital schistosomiasis
Source: PLoS Negl Trop Dis. 2022 Jul 5;16(7):e0009995. doi: 10.1371/journal.pntd.0009995 (PMC9286223; doi:10.1371/journal.pntd.0009995)
Supplement: Attachment — Submitted filename: Response to Reviewers 29.04.2022.docx [file pntd.0009995.s002.docx]

**Response to Reviewers**

Methods

Reviewer #1:

1)      Line 288 – 290. It seems interesting that the mean RPC count was statistically different between reviewers A and B and also A and C for the RPC count when the numbers were so small (0-3, 0-3, and 0-4). Since these numbers are so low, is there another way to compare them that would be more meaningful?

**We identified calculation mistakes in the comparison of CL and RP scores between raters. Using the Kruskal-Wallis test we found that there was no statistically significant difference between the three raters. Hence, no inter-rater differences should be observed. The mistakes were caused by lacking correction for pairwise comparison when using the Wilcoxon test to test the inter-rater difference. After the mistakes were corrected no statistically significant inter-rater differences were observed in the comparison of CL scores and RP scores. The manuscript has been adjusted. Line 233-234; 281-289; 296-302 and 364-368.**

2)      Line 291-292. I am not sure how the median RPC could be significantly different in step 2a and 2b can be different when they are both 1? “Median RPC (IQR) was 1 (0-3) in Step 2a and 1 (0-2) in Step 2b, thus statistically significant (p=0.002)”

**Please see the answer to 1).**

3) Lines 218-223: When the impact of the image quality on the CLP rating was assessed between the reviewers in separate sessions, the authors say that the images were stratified to whether they were depicted optimally or sub-optimally? I see later in line 264 – 265 that the reviewers felt that the quality of all the images was adequate for CLP and RPC determination? So could I clarify that ultimately no images were stratified to the group that was sub-optimally depicted?
**In the 27 images with partial visible cervix, at least 80% of the cervix was visible. All suboptimal images were reviewed in order to determine if the non-visible part could be important for the analysis. The non-visible part was always located in the outer circumference of the cervix and only a small fraction was involved. We therefor included all images in the final analysis.**

**Line 270 has been changed.**

4) Could I clarify that while all observers had access to the same images, each observer could choose to rate/view the images at different zoom settings?

**That is correct. The FGS QubiFier allows the reviewers to place the grid at any zoom ensuring the at grid covers the entire the whole circumference of the cervix. When the grid is placed correctly it will be locked so that it follows the image of the cervix when zoom is applied. The reviewers were allowed to zoom in the image after the grid has been placed so that even small details could be detected. Of cause a difference in zoom level when reviewing could result in discrepancy. For future studies a fixed zoom level could be possible but on the contrary a zoom level appropriate for reviewing one images is maybe not sufficient for reviewing another image. As described in line 309 one of the major reasons for discrepancy among the reviewers was difference in zoom of the cervix when the grid was placed and not lesions being overlooked.**

**Changes have been made in line 168-170 and 215-216.**

--------------------
Results

Reviewer #1:

The Figures are a nice addition, but Figure 2 and 3 were challenging to access due to the text size.
1)      Figure 1 – This is a nice figure. Unfortunately, the text under the “qubifier” heading is too small to read

**The text size has been changed from #10 to #12**

2)      Figure 3 – Unfortunately the labeling of the axes is too small and blurry to read

**This has been corrected**

--------------------
Conclusions

Reviewer #1:

Yes, overall the conclusions are supported by the data presented. The limitations of the analysis are clearly described. The authors discuss how the data can be helpful to advance the field of FGS.I do still have concerns about how two of the FGS lesions are still presented in the conclusion as being pathognomonic, without adequate data to support this conclusion. Given that FGS lesions lack specificity, I think it is challenging to call any of the four lesions “pathognomonic” (even rubbery papules or grainy sandy patches).

For example, in the discussion line – 326 – 327 the authors say “it seems reasonable to categorize grainy sandy patches and rubbery papules as FGS pathognomonic lesions.” Where in the manuscript have the authors provided evidence to support this statement? Since rubbery papules can look like Nabothian cysts, thus I think this position will be challenging to support (I do acknowledge that in Randrianosolo 2015 that 100% of the rubbery papules biopsied contained eggs [Table 2], but this is a small number). Additionally, in Kjetland’s 2005 “Simple clinical manifestations” paper, if I have read the tables correctly, didn’t only 30-60% of women with grainy sandy patches have eggs in any genital specimen vs biopsy (Table 3)? I am not sure this evidence is compelling enough to consider the grainy sandy patches pathognomonic either. Please consider either removing this statement, or providing additional evidence. If there is compelling evidence to cite, please could the authors consider adding this at line 185 where the four lesions are described.

I have still found a few other instances where FGS lesions are referred to as “pathognomonic”.

**The word pathognomonic has been deleted from the text (line 133, 182, 185, 338, 358)**

b.      Discussion line 345 – 346 In this sentence the authors describe the rubbery papule as a “fgs pathognomonic lesion”. Please rephrase.

**See above**

c.      Line 133 – In the inclusion criteria – there is a reference to pathognomonic FGS lesions, please rephrase.
**See above**

d.      Line 185 – "types of pathognomonic lesions" please rename this section to be congruent with the changes made in the last revisions (Types of FGS associated cervical changes/lesions etc)

**See above**
--------------------
Editorial and Data Presentation Modifications?
Reviewer #1:

1) Line 46 - one type of FGS associated lesions (Please check plurals, should this be lesion?)
**Corrected (line 44)**

2)      Line 51-53 - I think the last few sentences of the abstract might need to be clarified. Currently, the way the abstract is worded (in regards to the natural history of egg-induced pathology and assessing praziquantel treatment efficacy) it almost seems like these measures will be evaluated in this manuscript. Perhaps adjusting the wording to clarify this will be a future endeavor would be helpful here, such as: “In the future, grading of cervical pathology by CLP may provide insight into the natural course of schistosome egg-induced pathology of the cervix and may have a role in assessing praziquantel treatment efficacy against FGS.”

**Thank you, has been amended (line 50-55)**
3)      Lines 62- 74 - Author Summary: This segment gives a nice summary about FGS and the future uses of this technology but I am not sure that this segment tells the reader what the manuscript is about. I think the main aim was “The aim of the study was to test inter- and intra-rater reliability using a cervical lesion proportion (CLP) measure in a series of digital images from women living in a *Schistosoma haematobium* hyperendemic area in Madagascar”. Perhaps this could be brought into the author summary?

**Thank you, again – appreciated and amended (line 67-70),**
4)      Lines 85 – 89. The content of these sentences is challenging to access because the sentence is so long. Could it potentially be broken up with a period (potentially between the “anastomoses in the pelvic region” and “eggs”?

**This is good suggestion. Revised accordingly (line 84).**

5)      Please review lines 88 and 92 for sentence fragments.

**Revised (line 87-90)**

6)      Line 92-93. Could the sentence containing the “poor quality of sexual life” potentially be rephrased to express a more objective concept? I think the authors are saying that FGS may have a negative impact on the woman’s sexual experience, but the current phrasing feels somewhat subjective. The authors could also consider removing this phrase.

**Agree. Revised (line 91-92)**

7)      Schistosoma should be italicized throughout – please review lines 98, 324, 331, 393, 402
**Corrected**

8)      Line 113 – “imperatively needed” feels redundant to me – could one word or the other suffice?
**“Imperatively” removed**

9)      Line 167: superimposed?
**Corrected**

10)     Line 344: validation (Since this is not a validation study, do the authors mean assessment?)
**Corrected**

11)     Lines 323-326 – please provide a reference.
**Added**

12)     Lines 378-379: I’m not sure what you mean by “as mobile phones with camera features have pioneered the African continent” and I’m wondering if this particular portion of the sentence is necessary? And if necessary, should this be referenced?

**Removed**
--------------------
Summary and General Comments
Reviewer #1:

The revised manuscript is much clearer and is easier to follow. Thank you for the helpful revisions.

I do think that the wording around the association between FGS and HIV could be clarified by the authors. I believe there has only been one study suggesting an association with HIV transmission and schistosomiasis in women and this was a retrospective study in participants with Schistosoma antibodies (the participants in this study did not have FGS evaluations) (PMID 30543654). Thus, I think it is not accurate to state that FGS is considered a risk factor for HIV transmission.

I think it is also important to be clear regarding the difference between the association between schistosomiasis/FGS and HIV. At line 94, the authors reference a meta-analysis by Patel et al. The abstract for the Patel article clearly says “A significant association of schistosomiasis with HIV was found, however, a specific summary estimate for FGS could not be generated.” If an association with HIV is discussed, based on the Patel reference (12), I am not sure that it is correct to make the leap to FGS and then again to HIV transmission. Reference 11 is a hypothesis piece and can't stand alone to support the association between FGS and HIV transmission.

a.      Line 66 (Author Summary). I think it is still an overstatement to say that “FGS is considered as a risk factor for transmission of HIV” – please rephrase.
**Changed to hypothesized**

b.      Line 94. I still think it is an overstatement to say “FGS is suspected to be contributing risk factor for transmission of human immunodeficiency virus (HIV) in Schistosoma endemic areas of Sub-Saharan Africa”. Please rephrase.
**Changed to hypothesized**
